# Investigating the Validity of the Australian Early Development Census

**DOI:** 10.1007/s10578-023-01502-3

**Published:** 2023-03-05

**Authors:** Sarah Howells, Ha Trong Nguyen, Sally Brinkman, Francis Mitrou

**Affiliations:** 1 Education Futures, University of South Australia, Adelaide, SA 5000 Australia; 2 Telethon Kids Institute, Perth, Australia; 3 Centre for Child Health Research, The University of Western Australia, Perth, WA Australia

**Keywords:** Child development, Construct validity, Well-being, Young children

## Abstract

This article continues evaluation of the construct validity of the Australian Early Development Census (AEDC) through comparison with linked data from a sample of 2216 4–5 year old children collected as part of the Longitudinal Study of Australian Children (LSAC). This builds on the construct validity assessment of Brinkman et al*.* (Early Educ Dev 18(3):427–451, 2007) based on a smaller sample of linked Australian Early Development Instrument (AvEDI) and LSAC children, in which moderate to large correlations were apparent between teacher-rated AvEDI domains and subconstructs and LSAC measures, with lower levels apparent for parent reported LSAC measures. In the current study, the data showed moderate to low correlations between the domains and subdomains from the AEDC and teacher reported LSAC data. Differences in testing times, data sources (e.g. teachers versus carers) and levels of exposure to formal schooling at the time of testing are all discussed to account for the observed outcomes.

## Introduction

The period of early childhood has been identified as foundational for future health, socioeconomic and emotional success [1, 2]. Developmental level at school-entry particularly has been found to be predictive of future capacity for learning, and socioeconomic attainment [3]. A range of indicators have been identified for early childhood which allow for assessment of current and future levels of development. The Australian Early Development Census (AEDC) collects data using the Australian version of the Early Development Instrument (AvEDI).^1^ Australia is the only high income country to collect developmental data for children nationally at the time of entry to full-time school [4]. It is a revision of the Canadian Early Development Instrument (EDI), a teacher-completed measure of school readiness assessed through five domains: Physical health and wellbeing, Social competence, Emotional maturity, Language and cognitive development, and Communication skills and general knowledge [5, 6]. The AEDC enables a population level assessment of the key aspects of child development within a single instrument and yields data with the potential for benchmarking on universal child development approaches [7, 8]. Testing of the AvEDI using the Rasch model demonstrated high reliability and validity [9, 10], validity has been demonstrated in both Canada and Australia [11], and it has been shown to be predictive of later educational outcomes [12]. Validity of the AvEDI has been further explored against independently reported datasets. A 2007 study by Brinkman et al*.* [9] examined AvEDI validity using a nested study comparing AvEDI results from 642 children against contemporaneous data from the Longitudinal Study of Australian Children (LSAC), a nationally representative cross-sectional sample of Australian children [13]. The study reviewed the consistency of correlations of early child development measures between the AvEDI subconstructs and independently reported multimethod measures collected contemporaneously by the LSAC. Correlations between AvEDI domains and subconstructs were found to be moderate to high for LSAC measures based on teacher-report, but lower when compared to parent-completed or interview-directly assessed measures. Several factors were proposed to account for the lack of stronger correlations between the datasets. These include variation in the ages of the children and their relative exposure to formal primary education at the time of data collection within the two datasets. AvEDI discriminant validity was suggested by the consistency of lower correlations between constructs which were conceptually different.

In the current study, we proposed further validity testing of the AvEDI against a larger and more age appropriate^2^ LSAC sample than has previously been available. We hypothesise that this sample will provide increased strength or correlations for validity measures. We use linked AEDC/LSAC data to provide empirical evidence on the validity of the AEDC.

## Materials and Methods

The LSAC is a biennial nationally representative clustered cross-sequential sample of two cohorts of Australian children: the first group from all children born between March 2003 and February 2004 (Birth or B-Cohort), and the second from all children born between March 1999 and February 2000 (Kindergarten or K-Cohort) [14]. The initial sample of B-Cohort consisted of 5107 infants aged 0–1 in 2004, and K-Cohort 4983 children aged 4–5 years in 2004. The LSAC was initiated in 2004 and the latest available release was surveyed in 2020 when B (K) cohort children were 16–17 (20–21) years old. LSAC explored 3 domains, Health and physical development, Social and emotional functioning, and Learning and cognitive ability [15]. Health and physical development explored general health and medical conditions, nutrition and motor skills. Social and emotional functioning measures mental health, relationships, and social and emotional well-being. Learning and cognitive development looks at academic skills and outcomes, numeracy and literacy. The survey collected data on early development, health, education and living situation, from a variety of sources: teacher/caregiver completed questionnaires, parent self-completed questionnaires, face-to-face parent interviews, interviewer observation, and direct child observation. Assessments were based on well-established tools including Parent’s Evaluation of Developmental Status (PEDS), Pediatric Quality of life Inventory (PedsQL) [16], Short Temperament Scale for Children (STSC), Strengths and Difficulties Questionnaire (SDQ) [17], Peabody Picture Vocabulary Test (PPVT), Who am I? (WAI), and additional measures such as teacher rating of literacy and numeracy skills and rating of peer relationship quality [9]. Nationwide, 1976 postcodes were stratified by state, territory and capital city statistical division to ensure the sample was representative of the target population. Postcodes with less than 20 children were excluded from the survey. The current study used data from the B-Cohort collected during Wave 3, which overall consisted of 4386 4–5 year old children (88.2% response rate) transitioning to school [18]. Wave 3 data was collected from March 2008 to February 2009.

The Australian Early Development Census has collected data every 3 years since 2009, and measures developmental outcomes for children commencing their first year of full-time school through five key developmental domains [19].The Physical health and well-being domain measures children’s readiness for the school day, fine and gross motor skills, and physical independence. Social competence is measured overall, and also includes responsibility and respect, plus readiness to learn. The Emotional maturity domain measures prosocial behaviour and willingness to help, plus behaviours concerning anxiety, fear, aggression, hyperactivity, and inattention. The domain of Language and cognitive skills measures school-based basic and advanced literacy, basic numeracy, and interest in literacy, numeracy and memory tasks. The final domain, Communication skills and general knowledge, assesses these features in terms of broad developmental skills. The domains are assessed through 96 core questions, assessed using either a yes/no or Likert scale response. Children receive a score between 0 and 10 for each domain, with higher scores indicating a higher level of development. Context for the child is provided through collection of details such as the child’s care arrangements, participation in early education programs prior to school, and demographic and geographical information. The instrument is completed by the primary classroom teacher based on at least one month’s knowledge of the child. AvEDI is not an individual diagnostic instrument but a tool to assess development of children according to a range of developmental domains. Individual scores are de-identified and reported as data aggregated by group (class, school, neighbourhood, province/state, country).

The current study is based on a subset of the 2009 AEDC collection cycle, the latter of which involved 261,147 children, representing 97.5% of the eligible child population. AEDC data was only available for that subset of B-cohort children (n = 2459) who took the AEDC and then consented to have their AEDC data linked to LSAC. This subset of the LSAC B-Cohort were 5–6 years of age at the time of the AEDC*.* AEDC data collection took place between 1st May and 31st July 2009. This linked LSAC-AEDC dataset forms the basis of our analysis. We used continuous scores for AEDC as per Brinkman et al. [9]. As AEDC is designed to capture “school readiness”, we use developmental outcomes recorded at LSAC wave 3 (or year 2008) when the B cohort children were 4–5 years of age, and before they started their formal school years.

As the constructs that the AEDC measures that relate to early child development and school readiness are latent, that is, not directly observable, their validity as a general measure of child development can only be assessed indirectly. As such, assessment of validity is based on establishing consistent correlations between the overall, domain and subdomain scores, and other measures of early child development.

Our key research question was whether a larger LSAC sample than was previously available allows for an improved calibration of the AEDC. Following Brinkman [9] we analysed the strength of association between each AEDC domain and the comparable LSAC measure using Spearman correlation coefficients. Correlational analyses were used to analyse nomological network of associations between the AEDC and the LSAC. Also, following Brinkman [9], we adopt the popular thresholds suggested by Hopkins [20] for pairwise correlation strength in which thresholds of 0.90, 0.70, 0.50, 0.30 and 0.0 are used to indicate a “very large”, “large”, “moderate”, “small” and “little or none” correlation, respectively.

**Fig. 1:**
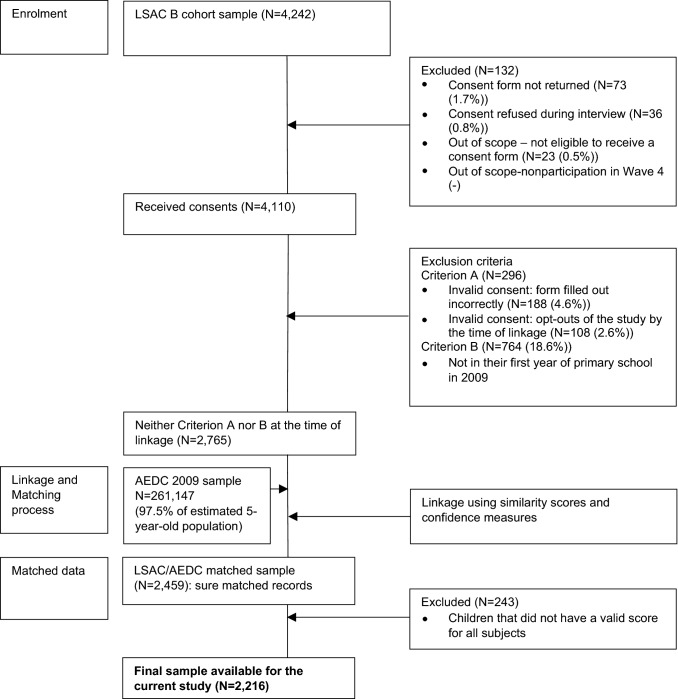
Sample participant flow diagram (MS Office)

The Participant Flow Diagram (Fig. 1) shows the linkage protocol and associated participant numbers for the linked LSAC/AEDC sample used in the current study, which resulted in a final matched sample of 2459 children. This follows the linkage process outlined in detail in Bandara et al*.* [21] which uses the child’s first name, surname, gender, date of birth, school name and school address as identifying variables. Some children were classified as being out of scope, or ineligible to receive a consent form. Out of scope exclusions included home schooling, special care needs/intellectual disability, or distance education. Using the matched sample, we make a further restriction that matched children have valid scores for all subjects. This restriction led to a final sample of 2216 children.

The comparative demographic detail of included and excluded children in provided in Appendix Table 8. As the number of linked children excluded from our study due to incomplete AEDC scores was small (243), they were included with unlinked children in this table. Table 8 suggests that included children were statistically significant more likely to come from families with higher maternal education (mother with a postgraduate degree), be living with both parents and/or living in own home, or live in an area where a higher percentage of the community are working. This suggests that linked children were of overall higher socioeconomic status than unlinked children. With excluded children being more likely to come from a non-English-speaking background and have younger mothers, the patterns were similar to children from non-responding families in the subsequent rounds of LSAC [18].

The demographics of the sample are provided in Appendix 2. The average age of the AEDC sample (5.17 years) was slightly older than the LSAC sample (4.75). The sample was evenly divided by gender, with 52% males. The majority of children did not identify as being Aboriginal or Torres Strait Islander (97%). Some 10% of children had mothers who were from a non-English speaking background (NESB) while 8% had mothers born overseas in an English-speaking background (ESB) country. Approximately three-quarters of children had been breastfed at early childhood (74%) and lived in non-rental accommodation (77%). The majority of children (89%) lived with both parents and had on average one-two siblings. Approximately two thirds of children had mothers with either a diploma or certificate (39%) or postgraduate degree (38%).

Following Brinkman et al. [9], we employ a range of child development outcomes as recorded in LSAC which map to the five AEDC domains. Moreover, to make the scaling consistent across comparable measures between LSAC and AEDC, where necessary, we rescale some LSAC measures in such a way that a higher score indicates a more desirable outcome. Table 1 shows the number of children with valid data, and the mean, standard error, median, and minimum and maximum values for each variable. The difference between N for each measure and the total number of participants indicates any missing data for each LSAC outcome for this matched sample.

**Table 1:** Descriptive statistics for LSAC variables included in the analysis (n = 2216)

AED domain	LSAC measure	N	Mean	SD	Median	Min	Max
Physical health & well-being	Global health (P1–Rev.)	2216	4.46	0.72	5	1	5
	PedsQL physical health summary (P1)	1983	85.43	9.88	87.5	17.86	100
	Fine motor skills (TC–Rev.)	1726	3.10	0.61	3	1	4
	Gross motor skills (TC- Rev.)	1730	3.09	0.51	3	1	4
Social competence	PedsQL social functioning (P1)	1979	85.38	12.97	90	15	100
	STSC sociability (P1–Rev.)	1987	3.66	1.43	4	1	6
	SDQ prosocial (P1)	1987	7.79	1.71	8	1	10
	SDQ peer problem (P1–Rev.)	1987	8.74	1.35	9	1	10
	SDQ conduct (P1–Rev.)	1987	7.93	1.73	8	0	10
	Child separation behaviour (TC)	1706	3.43	0.42	3.5	1.38	4
	Child reunion behaviour (TC)	1711	3.51	0.36	3.57	2	4
	SDQ prosocial (TC)	1731	7.33	2.23	8	0	10
	SDQ peer problem (TC–Rev.)	1731	8.76	1.54	9	0	10
	SDQ conduct (TC–Rev.)	1731	8.97	1.78	10	0	10
Emotional maturity	SDQ prosocial (P1)	1987	7.79	1.71	8	1	10
	SDQ hyperactivity (P1–Rev.)	1986	6.89	2.03	7	0	10
	SDQ emotional symptoms (P1)	1987	8.64	1.44	9	2	10
	STSC reactivity (P1)	1987	2.55	0.82	2.5	1	6
	PedsQL emotional functioning (P1)	1983	75.09	13.71	75	16.67	100
	SDQ prosocial (TC)	1731	7.33	2.23	8	0	10
	SDQ hyperactivity (TC–Rev.)	1730	7.95	2.26	9	0	10
	SDQ emotional symptoms (TC–Rev.)	1730	9.09	1.45	10	1	10
	Warm relationship (TC)	1731	4.44	0.62	4.67	1.33	5
	Conflict/anger (TC–Rev.)	1731	4.58	0.67	4.86	1.14	5
Language & cognitive development	PEDS expressive language concern (P1–Rev.)	2215	0.77	0.42	1	0	1
	PEDS receptive (P1–Rev.)	2216	0.96	0.20	1	0	1
	Speech therapy via school (P1–Rev.)	2216	0.89	0.31	1	0	1
	Reading competencies scale (P1)	2216	0.43	0.71	0	0	3
	Home activities (P1)	2216	1.71	0.55	1.71	0	3
	Reading competencies (TC)	1726	1.81	0.85	2	0	5
	Writing competencies (TC)	1728	3.33	1.34	4	0	6
	Numeracy competencies (TC)	1719	3.59	1.13	4	0	5
	PPVT (ITV)	2188	65.48	5.83	66.19	34.19	84.78
	WAI (ITV)	2153	64.67	7.33	65	29.94	90.41
Communication skills & general knowledge	Reading competencies (P1)	2216	0.43	0.71	0	0	3
	Open communication scale (TC)	1731	4.38	0.70	4.67	1	5
	Reading competencies (TC)	1726	1.81	0.85	2	0	5
	Writing competencies (TC)	1728	3.33	1.34	4	0	6
	Numeracy competencies (TC)	1719	3.59	1.13	4	0	5
	PPVT (ITV)	2188	65.48	5.83	66.19	34.19	84.78
	WAI (ITV)	2153	64.67	7.33	65	29.94	90.41

P1: reported by Parent 1; TC: reported by Teacher; ITV: assessed by Interviewer. “Rev.” refers to reversed scaling. “Global health” is constructed from responses to a question asking the corresponding parent: “In general, how would you say your child's current health is?: 1 Excellent; 2 Very good; 3 Good; 4 Fair; 5 Poor”. “Fine motor skills” is constructed from responses to a question asking teacher “Rate how this child has compared with other children of a similar age, over the past few months: 1 More competent than others; 2 As competent as other children; 3 Less competent than others; 4 Much less competent”. “Gross motor skills” is calculated similarly. “PEDS expressive language concern” is constructed from responses to a question asking “Do you have any concerns about how the study child talks and makes speech sounds?: 1 No; 2 Yes; 3 A little”. “PEDS receptive” is constructed from responses to a question asking “Do you have any concerns about how the study child understands what you say to him/her?: 1 No; 2 Yes; 3 A little”. “Speech therapy via school” is constructed from responses to a question asking “In the last 12 months, have you used any of these services for the study child? Speech therapy”

*PEDS* Parent's Evaluation of Developmental Status; *PedsQL* Pediatric Quality of Life Inventory; *STSC* Short Temperament Scale for Children; *SDQ* Strengths and Difficulties Questionnaire; *PPVT* Peabody Picture Vocabulary Test; *WAI* Who Am I?

## Results

Table 2 shows the correlation between the AvEDI instruments. All correlations were statistically significant at 0.1%.

**Table 2:** Correlation among AEDC measures

	Physical readiness for school day	Physical independence	Gross & fine motor skills	Overall social competence	Responsibility & respect	Approaches to learning	Readiness to explore new things	Prosocial & helping behaviour	Anxious & fearful behaviour	Aggressive behaviour	Hyperactive & inattentive behaviour	Basic literacy	Interest in literacy/numeracy & memory	Advanced literacy	Basic numeracy	Communication skills & general knowledge
Physical readiness for school day	1.00															
Physical independence	0.20	1.00														
Gross & fine motor skills	0.43	0.33	1.00													
Overall social competence	0.33	0.19	0.47	1.00												
Responsibility & respect	0.34	0.19	0.34	0.72	1.00											
Approaches to learning	0.38	0.25	0.55	0.64	0.69	1.00										
Readiness to explore new things	0.23	0.11	0.37	0.37	0.35	0.48	1.00									
Prosocial & helping behaviour	0.24	0.13	0.31	0.55	0.54	0.53	0.39	1.00								
Anxious & fearful behaviour	0.24	0.17	0.29	0.43	0.28	0.38	0.24	0.31	1.00							
Aggressive behaviour	0.28	0.12	0.18	0.54	0.72	0.46	0.18	0.41	0.26	1.00						
Hyperactive & inattentive behaviour	0.32	0.18	0.32	0.54	0.71	0.69	0.26	0.43	0.31	0.68	1.00					
Basic literacy	0.23	0.21	0.34	0.26	0.25	0.43	0.29	0.28	0.16	0.18	0.28	1.00				
Interest in literacy/numeracy & memory	0.22	0.20	0.36	0.31	0.29	0.52	0.40	0.27	0.20	0.17	0.35	0.52	1.00			
Advanced literacy	0.20	0.16	0.37	0.31	0.26	0.42	0.29	0.32	0.14	0.18	0.27	0.57	0.41	1.00		
Basic numeracy	0.21	0.20	0.37	0.29	0.24	0.45	0.30	0.21	0.16	0.15	0.27	0.60	0.57	0.48	1.00	
Communication skills & general knowledge	0.31	0.22	0.64	0.52	0.37	0.60	0.45	0.38	0.29	0.18	0.31	0.43	0.46	0.43	0.47	1.00

Spearman correlation coefficients

All correlations are statistically significant at 0.1%

### Construct Validity Findings

#### AvEDI Physical Health and Well-being Domain (Table 3)

**Table 3:** Correlations among the AEDC and LSAC physical health and well-being measures

	Global health (P1–Rev.)	Peds QL physical health summary (P1)	Fine motor skills (TC–Rev.)	Gross motor skills (TC- Rev.)	Physical readiness for school day (AEDC)	Physical independence (AEDC)	Gross & fine motor skills (AEDC)
Global health (P1–Rev.)	–						
Peds QL physical health summary (P1)	0.22**	–					
Fine motor skills (TC–Rev.)	0.03	0.05*	–				
Gross motor skills (TC- Rev.)	0.05*	0.05*	0.32**	–			
Physical readiness for school day (AEDC)	0.04	− 0.01	0.09**	0.08**	–		
Physical independence (AEDC)	0.05*	0.01	0.13**	0.12**	0.20**	–	
Gross & fine motor skills (AEDC)	0.08**	− 0.01	0.26**	0.19**	0.43**	0.33**	–

Spearman correlation coefficients

The symbol *denotes significance at the 5% level and ** at the 1% level

P1: reported by Parent 1; TC: reported by Teacher. “Rev.” refers to reversed scaling

The LSAC Peds QL Physical health summary was weakly correlated with the AEDC Physical health and well-being domain. Small positive correlations were apparent between the LSAC teacher-rated Fine motor skills and Gross motor skills and the AEDC Gross and fine motor skills subdomain (0.26 and 0.19). LSAC teacher-rated Fine motor skills and Gross motor skills also correlated positively and only weakly with the AEDC Physical independence score (0.13 and 0.12). This suggests that different skills were being measured in these components of the LSAC and AEDC.

#### AEDC Social Competence Domain (Table 4)

**Table 4:** Correlations among the AEDC and LSAC social competence measures

	PedsQL social functioning (P1)	STSC sociability (P1–Rev.)	SDQ prosocial (P1)	SDQ peer problem (P1–Rev.)	SDQ conduct (P1–Rev.)	Child separation behaviour (TC)	Child reunion behaviour (TC)	SDQ prosocial (TC)	SDQ peer problem (TC–Rev.)	SDQ conduct (TC–Rev.)	Overall social competence (AEDC)	Responsibility & respect (AEDC)	Approaches to learning (AEDC)	Readiness to explore new things (AEDC)
PedsQL social functioning (P1)	–													
STSC sociability (P1–Rev.)	0.10**	–												
SDQ prosocial (P1)	0.21**	0.10**	–											
SDQ peer problem (P1–Rev.)	0.38**	0.10**	0.24**	–										
SDQ conduct (P1–Rev.)	0.24**	-0.02**	0.32**	0.23**	–									
Child separation behaviour (TC)	0.12**	0.19	0.13**	0.16**	0.06**	–								
Child reunion behaviour (TC)	0.07**	− 0.08**	0.10**	0.08**	0.11*	0.38**	–							
SDQ prosocial (TC)	0.12**	0.00**	0.23**	0.17**	0.16**	0.32**	0.35**	–						
SDQ peer problem (TC–Rev.)	0.14**	0.03	0.08**	0.27**	0.04**	0.38**	0.22**	0.37**	–					
SDQ conduct (TC–Rev.)	0.10**	− 0.11	0.16**	0.11**	0.22	0.18**	0.39**	0.52**	0.26**	–				
Overall social competence (AEDC)	0.11**	− 0.02**	0.14**	0.15**	0.16**	0.14**	0.16**	0.29**	0.19**	0.31**	–			
Responsibility & respect (AEDC)	0.04**	− 0.11	0.13**	0.06**	0.17**	0.07**	0.15**	0.30**	0.12**	0.36**	0.72**	–		
Approaches to learning (AEDC)	0.05	− 0.07**	0.11**	0.12**	0.12**	0.12**	0.15**	0.27**	0.16**	0.23**	0.64**	0.69**	–	
Readiness to explore new things (AEDC)	0.03*	0.09**	0.07**	0.07**	0.06**	0.11**	0.02**	0.10**	0.06**	0.03**	0.37**	0.35**	0.48**	–

Spearman correlation coefficients

The symbol *denotes significance at the 5% level and ** at the 1% level

P1: reported by Parent 1; TC: reported by Teacher

*Rev*. refers to reversed scaling

A weak correlation was observed between LSAC teacher-rated Prosocial scale and the AEDC Overall social competence scale (0.29) and Approaches to learning scale (0.27). Weak positive correlations were observed between the LSAC teacher-rated Conduct scale and the AEDC Overall social competence scale, and Responsibility and respect and Approaches to learning subdomain scores (0.31, 0.36 and 0.23). The LSAC teacher-rated Child separation behaviour was weakly positively correlated with the AEDC Overall social competence, Approaches to learning and Readiness to explore new things (0.14, 0.12 and 0.11). There was no or little correlation between LSAC parent-reported PedsQL Social functioning, STSC Sociability, and SDQ Prosocial, Peer problem and Conduct scores and the AEDC Social competence measures. This suggests that the AEDC measures social competency through different characteristics than is captured through the LSAC tools.

#### AEDC Emotional Maturity Domain (Table 5)

**Table 5:** Correlations among the AEDC and LSAC emotional maturity measures

	SDQ prosocial (P1)	SDQ hyperactivity (P1 − Rev.)	SDQ prosocial (P1)	STSC reactivity (P1)	PedsQL emotional functioning (P1)	SDQ prosocial (TC)	SDQ hyperactivity (TC − Rev.)	SDQ emotional symptoms (TC − Rev.)	Warm relationship (TC)	Conflict/anger (TC − Rev.)	Prosocial & helping behaviour (AEDC)	Anxious & fearful behaviour (AEDC)	Aggressive behaviour (AEDC)	Hyperactive & inattentive behaviour (AEDC)
SDQ prosocial (P1)	–													
SDQ hyperactivity (P1–Rev.)	0.30**	–												
SDQ emotional symptoms (P1)	0.09**	0.15**	–											
STSC reactivity (P1)	− 0.32**	–0.30**	–0.23**	–										
PedsQL emotional functioning (P1)	0.16**	0.18**	0.43**	− 0.35**	–									
SDQ prosocial (TC)	0.23**	0.17**	0.03	− 0.13**	0.05	–								
SDQ hyperactivity (TC −Rev.)	0.19**	0.30**	0.00	− 0.14**	0.02	0.53**	–							
SDQ emotional symptoms (TC–Rev.)	0.07**	0.01	0.17**	− 0.04	0.09**	0.14**	0.18**	−						
Warm relationship (TC)	0.13**	0.04	0.06*	− 0.03	0.03	0.45**	0.24**	0.18**	–					
Conflict/anger (TC −Rev.)	0.15**	0.18**	− 0.01	− 0.12**	0.08**	0.47**	0.56**	0.24**	0.31**	–				
Prosocial & helping behaviour (AEDC)	0.14**	0.14**	0.07**	− 0.09**	0.05*	0.27**	0.25**	0.10**	0.17**	0.22**	–			
Anxious & fearful behaviour (AEDC)	0.11**	0.09**	0.09**	− 0.04	0.06**	0.15**	0.14**	0.19**	0.08**	0.15**	0.31**	–		
Aggressive behaviour (AEDC)	0.11**	0.20**	− 0.04*	− 0.13**	0.03	0.28**	0.34**	− 0.01	0.07**	0.38**	0.41**	0.26**	–	
Hyperactive & inattentive behaviour (AEDC)	0.12**	0.24**	− 0.07**	− 0.12**	0.00	0.28**	0.42**	0.01	0.08**	0.34**	0.43**	0.31**	0.68**	–

Spearman correlation coefficients

The symbol *denotes significance at the 5% level and ** at the 1% level

P1: reported by Parent 1; TC: reported by Teacher

*Rev*. refers to reversed scaling

A weak correlation was seen between LSAC teacher rated Prosocial scale and AEDC Pro-social and helping behaviour scale, the Aggressive behaviour score and the Hyperactive and inattentive behaviour score (0.27, 0.28 and 0.28). Moderate positive correlations were observed between the LSAC teacher-rated Hyperactivity scale and the AEDC Aggressive behaviour scale and Hyperactive and inattentive behaviour scale (0.34 and 0.42). The LSAC teacher rated Conflict/anger scale was also moderately weakly correlated with the AEDC Aggressive behaviour scale and the Hyperactive and inattentive behaviour scale (0.38 and 0.34). Little or no correlation was observed between the LSAC parent-rated Emotional maturity measures and the AEDC Emotional maturity subdomain scores.

#### AEDC Language and Cognitive Development Domains (Table 6)

**Table 6:** Correlations among the AEDC and LSAC language and cognitive development measures

	PEDS expressive language concern (P1 −Rev.)	PEDS receptive (P1 −Rev.)	PEDS expressive language concern (P1 −Rev.)	Reading competencies scale (P1)	Home activities (P1)	Reading competencies (TC)	Writing competencies (TC)	Numeracy competencies (TC)	PPVT (ITV)	WAI (ITV)	Basic literacy (AEDC)	Interest in literacy/numeracy & memory (AEDC)	Advanced literacy (AEDC)	Basic numeracy (AEDC)
PEDS expressive language concern (P1 −Rev.)	–													
PEDS receptive (P1–Rev.)	0.22**	–												
Speech therapy via school (P1–Rev.)	0.50**	0.17**	–											
Reading competencies scale (P1)	0.07**	0.01	0.08**	–										
Home activities (P1)	0.03	0.05*	0.00	0.09**	–									
Reading competencies (TC)	0.08**	0.06**	0.07**	0.19**	0.08**	–								
Writing competencies (TC)	0.12**	0.05*	0.09**	0.14**	0.03	0.55**	–							
Numeracy competencies (TC)	0.11**	0.06*	0.09**	0.15**	0.10**	0.44**	0.53**	–						
PPVT (ITV)	0.07**	0.09**	0.05*	0.08**	0.16**	0.14**	0.17**	0.2**	–					
WAI (ITV)	0.13**	0.08**	0.11**	0.27**	0.10**	0.32**	0.46**	0.36**	0.31**	–				
Basic literacy (AEDC)	0.09**	0.05**	0.06**	0.08**	0.07**	0.15**	0.24**	0.25**	0.25**	0.31**	–			
Interest in literacy/numeracy & memory (AEDC)	0.09**	0.08**	0.08**	0.08**	0.04*	0.15**	0.23**	0.22**	0.21**	0.26**	0.52**	-		
Advanced literacy (AEDC)	0.08**	0.05*	0.07**	0.18**	0.03	0.21**	0.30**	0.29**	0.20**	0.39**	0.57**	0.41**	–	
Basic numeracy (AEDC)	0.11**	0.08**	0.06**	0.09**	0.04	0.17**	0.26**	0.26**	0.26**	0.32**	0.60**	0.57**	0.48**	–

Spearman correlation coefficients

The symbol *denotes significance at the 5% level and ** at the 1% level

P1: reported by Parent 1; TC: reported by Teacher; ITV: assessed by Interviewer

*Rev*. refers to reversed scaling

Modest correlations were found between LSAC interviewer administered Who Am I? questionnaire and AEDC Basic literacy score, Advanced literacy score and Basic numeracy score (0.31, 0.39 and 0.32). Small correlations were found between LSAC teacher-rated Reading, Writing and Numeracy Competencies, PPVT score and the AEDC Literacy developmental subdomains. LSAC parent-reported PEDS Expressive language concern, Receptive and Expressive language concerns, Reading competencies, and Home activities scales and the AEDC Language and cognitive skills domain showed no correlation.

#### AEDC Communication Skills and General Knowledge Domains (Table 7)

**Table 7:** Correlations among the AEDC and LSAC communication skills and general knowledge measures

	Reading competencies scale (P1)	Open communication scale (TC)	Reading competencies (TC)	Writing competencies (TC)	Numeracy competencies (TC)	PPVT (ITV)	WAI (ITV)	Communication skills & general knowledge (AEDC)
Reading competencies scale (P1)	–							
Open communication scale (TC)	0.04	–						
Reading competencies (TC)	0.19**	0.22**	−					
Writing competencies (TC)	0.14**	0.23**	0.55**	–				
Numeracy competencies (TC)	0.15**	0.22**	0.44**	0.53**	–			
PPVT (ITV)	0.08**	0.15**	0.14**	0.17**	0.20**	–		
WAI (ITV)	0.27**	0.18**	0.32**	0.46**	0.36**	0.31**	–	
Communication skills & general knowledge (AEDC)	0.07**	0.22**	0.17**	0.25**	0.27**	0.27**	0.31**	–

Spearman correlation coefficients

The symbol *denotes significance at the 5% level and ** at the 1% level

P1: reported by Parent 1; TC: reported by Teacher; ITV: assessed by Interviewer

Weak to moderate correlations were found between LSAC interviewer administered PPVT and WAI and AEDC Communication skills and general knowledge scale (0.27 and 0.31). Weak correlations were found between the LSAC literacy and numeracy competencies scales and the AEDC Communication skills and general knowledge domain.

## Discussion

In 2007, Brinkman et al*.* [9] investigated the construct and concurrent validity of the Australian Early Development Index, an earlier version of AEDC. In Brinkman et al.’s study, the AvEDI data from 641 to 4–5 year old children were analysed in relation to other measures of early child development from the first wave of LSAC data for K-cohort in 2004, assessed by parent interview, direct assessment and teacher rating. Moderate to strong correlations were apparent between the AvEDI domains and subconstructs and the comparable LSAC measures based on teacher report. Less correlation was apparent when AvEDI domains were compared with parent reported LSAC items. Through the linkage of AEDC data to B-Cohort children from Wave 3, the current study afforded an opportunity to further assess validity based on a larger sample than was available in Brinkman et al. original study.

Overall, there was poor correlation between LSAC and AEDC scores in the current paper. The highest correlation, although only moderately strong, was between LSAC interviewer administered PPVT and WAI and AEDC Communication skills and general knowledge scales. The strongest association in Brinkman et al.’s analysis was similarly found in the areas of literacy and communication, although between LSAC teacher rated reading, writing and numeracy and AvEDI Language and cognition [9]. In the current study, moderate correlations were also found between the LSAC Who Am I? (WAI) interviewer administered questionnaire and AEDC Basic literacy score, Advanced literacy score and Basic numeracy score, again reflecting the strength of correlation in the literacy and numeracy domains. The WAI is a narrow construct measuring fine motor skills of drawing and copying [22], with suggestions that it assesses concepts of pre-literacy [23]. That the correlation was not stronger may be due to the simpler literacy concepts measured in the WAI, with copying and recognition of symbols, compared with the more sophisticated literacy and numeracy concepts tested in the AEDC, such as identify letters and sounds of the alphabet, awareness of rhyming words and counting to 20. Weaker correlations were observed across many measures such as LSAC Prosocial and AEDC Social competence scales, and LSAC Child separation behaviour and AEDC Overall social competence.

Little or no correlation was observed across various domains, such as the LSAC parent-rated Emotional maturity measures and the AEDC Emotional maturity subdomain scores. For instance, there was no or little correlation between LSAC parent reported PedsQL Social functioning or STSC Sociability, and AEDC Social competence measures. Similarly, no correlation was found between language competencies such as the LSAC parent reported PEDS Expressive language concern, Receptive and Expressive language scales and the AEDC Language domain. Positive moderate correlations were noted between LSAC and AEDC measures of hyperactivity and LSAC Conflict/anger and AEDC Aggressive behaviour scales. These scales use very similar items. For example, LSAC asks the teacher if the child “Often has temper tantrums or hot tempers” compared with the AEDC “Has temper tantrums”.

There was overall poor or no correlation between LSAC parent-report and AEDC measures. A similar relationship was noted both between parent- and teacher-reported scores within LSAC and in Brinkman et al.’s 2007 paper. Possible reasons for this will be discussed below.

### Limitations

Overall, this paper finds a lack of validity between the scales across LSAC and AEDC. This validation result while being specific to our case is furthermore not uncommon to the literature which typically finds it challenging to precisely capture skills, especially non-cognitive skills and among young children [24]. Previous research has shown that both the AEDC and LSAC have construct validity and provide a good indicator of child development at the population level [9, 11, 25]. Several factors may have however contributed to the generally low level or lack of correlation between the LSAC and AEDC datasets in our study.

The timing of testing of measures may have impacted on children’s scores. In the current study, the LSAC data was collected from April 2008 to April 2009, whereas the AvEDI data collection took place from May 2009 to July 2009. Thus, the children in the study were between 1 and 14 months older at the time of the AvEDI collection wave compared with the timepoint for LSAC data collection. Overall, the LSAC data are however based on children who are on average 5 months younger than the age at which Australian children have their AEDC accessed (i.e., on average, LSAC B cohort children in our sample were 5.2 years old at the time of AEDC). Many measures across domains may be expected to change quite significantly at this period of development. The weak positive correlations between fine and gross motor skills in the LSAC versus the AEDC, for example, may thus be due to the difference in the timing when LSAC and AEDC outcomes are measured. The LSAC data collection also occurred mainly prior to school entry, whereas the AEDC is collected after the start of formal schooling, whereby fine motor skills such as writing might be expected to be further developed.

Teacher report for the LSAC might be based on the child’s schoolteacher, or formal or informal preschool educator or carer. As the AEDC is based on teacher report, it is likely that a different educator provided the teacher reporting for many children in this analysis. The training and educational experience of the educator or carer may also differ considerably between LSAC and AEDC, resulting in potential variations in the reporting on the developmental outcomes of the child.

Furthermore, whilst children participating in the AvEDI had received at least one month of formal schooling, the LSAC (as represented by Wave 3, B cohort used here, not later iterations) is intended to primarily assess children prior to schooling and includes children in formal and informal care settings. Finally, in areas where there were low or absent correlations it is possible that the constructs were measuring different concepts. Thus, whilst some questions were very similar across the two surveys, other questions within the same subconstruct may have differed enough to capture different concepts overall. The previous validity of LSAC and AEDC demonstrates their value as measures of child development, with the current study nevertheless highlighting the complexity of the behaviours and developmental processes they are measuring. Various reasons were proposed for the poor correlation between the AEDC and LSAC linked data, however further research is required to explore the nature of the relationships between the datasets.

## Summary

This article continues evaluation of the construct validity of the Australian Early Development Census (AEDC) through comparison with linked data from a sample of 2216 4–5 year old children collected as part of the Longitudinal Study of Australian Children (LSAC). LSAC data on early child development was independently derived from multimethod measures derived approximately contemporaneous with the AEDC data collection. This builds on the construct validity assessments of Brinkman et al*.* [9] which was based on a smaller sample of 642 linked the Australian Early Development Instrument (AvEDI) and LSAC children in which moderate to large correlations were apparent between teacher-rated AvEDI domains and subconstructs and LSAC measures, with lower levels apparent for parent reported LSAC measures. In the current study, the data showed moderate to low correlations between the domains and subdomains from the AEDC and teacher reported LSAC data. We considered that factors such as the gap in the timing when LSAC and AEDC outcomes are measured, comparing data collected from parent versus teacher report, the measures utilised differing in the characteristics of child development being captured, and different levels of exposure to formal schooling at the time of testing were all likely to have impacted on the lower levels of correlation between the two measures. Overall, this paper finds a lack of validity between the scales across LSAC and AEDC. Given the previously recognised validity of the AEDC, further research is required to explore the apparent differences between the AEDC and LSAC data sets.

## Appendix 1

See Table 8

**Table 8:** Characteristics of included and non-included individuals

	Included	Excluded	Included–Excluded
	(1)	(2)	(3)
Child age (months)	57.03	58.22	− 1.187**
Male	0.52	0.51	0.008
Aboriginal	0.03	0.04	− 0.002
Low birth weight	0.05	0.06	− 0.002
Maternal age	35.59	34.97	0.622**
Mother NESB	0.10	0.14	− 0.035**
Mother ESB	0.08	0.09	− 0.005
Mother has a certificate or diploma	0.39	0.40	− 0.009
Mother has graduate or postgraduate degree	0.38	0.34	0.040**
Number of siblings	1.47	1.52	− 0.053
Live with both parents	0.89	0.83	0.052**
Breast fed at early childhood	0.74	0.69	0.047**
Lived in own home	0.76	0.67	0.091**
Home - % completed year 12 for linked area	47.63	46.94	0.690
Home - % family < $1 K/week in linked area	31.12	32.32	− 1.204**
Home - % working in linked area	63.35	62.80	0.548*
Metropolitan region	0.61	0.63	− 0.014
Home - % speak English in linked area	87.21	85.90	1.310**
Home - % Australian born in linked area	95.78	95.63	0.150
Home - % ATSI in linked area	2.25	2.54	− 0.285*
Number of observations	2216	2170	

Figures are sample means. Statistics are reported for a sample of B-cohort children who were surveyed in Wave 3. Tests are performed on the significance of the difference between the sample mean for included and excluded individuals

The symbol *denotes significance at the 5% level, and ** at the 1% level

## Appendix 2

See Table 9

**Table 9:** Final sample demographics

Variables	N	n	Mean	SD	Median	Min	Max
Child age at wave 3 survey time (years)	2216		4.75	0.2	4.75	4.17	5.75
Child age at the time of 2009 AEDC (years)	2216		5.17	0.38	5	5	6
Male	2216	1152	0.52	0.5	1	0	1
Aboriginal	2216	66	0.03	0.18	0	0	1
Low birth weight	2213	110	0.05	0.22	0	0	1
Maternal age (years)	2210		35.71	5.11	36	20	69
Mother NESB	2216	221	0.10	0.30	0	0	1
Mother ESB	2216	177	0.08	0.28	0	0	1
Mother has a certificate or diploma	2203	859	0.39	0.49	0	0	1
Mother has graduate or postgraduate degree	2203	837	0.38	0.48	0	0	1
Number of siblings	2216		1.47	0.92	1	0	7
Live with both parents	2216	1972	0.89	0.32	1	0	1
Breast fed at early childhood	2216	1639	0.74	0.44	1	0	1
Lived in own home	2216	1706	0.77	0.42	1	0	1

Statistics are reported for a sample of 2216 B-cohort children

“n” indicates the number of observations which have the characteristic mentioned in the first column

## Footnotes

1 In 2014, the Australian Early Development Index program was renamed the Australian Early Development Census (AEDC), with the data collection tool being renamed the Early Development Instrument rather than the Early Development Index.

2 In this current study, we observe actual AEDC scores so the actual ages that children have their AEDC scores evaluated. An earlier validation study by Brinkman et al*.* (2007) uses an earlier LSAC for K cohort with some pilot AEDI tests for about 642 4/5 year old children. As they noted, these ages are slightly younger than the age Australian children actually have their AEDC assessed.
